# The efficacy and safety of the *Shaoyao Shujin* tablet for knee osteoarthritis: study protocol for a randomized controlled trial

**DOI:** 10.1186/s13063-015-1121-3

**Published:** 2016-01-04

**Authors:** Xue-Wei Cao, Da Guo, Jin-Wen Liu, Wei Niu, Jun Liu, Jian-Ke Pan, Hui Xie, Wen-Wei Ouyang, Ding-Kun Lin

**Affiliations:** Department of Orthopedic Surgery, Guangdong Provincial Hospital of Traditional Chinese Medicine, 111 Dade Road, Guangzhou, Guangdong 510120 China; The Second School of Clinic Medicine, Guangzhou University of Chinese Medicine, 111 Dade Road, Guangzhou, Guangdong 510120 China; Department of Statistical Secretary, The Second School of Clinic Medicine, Guangzhou University of Chinese Medicine, 111 Dade Road, Guangzhou, Guangdong 510120 China

**Keywords:** Knee osteoarthritis, *Shaoyao Shujin* tablet, Clinical efficacy and safety, Randomized controlled trial

## Abstract

**Background:**

Knee osteoarthritis (KOA) is a major public health issue causing chronic disability as well as a burden on healthcare resources. In China, a herbal drug tablet has been used as an effective and conventional therapy to alleviate clinical symptoms caused by KOA. However, evidence gathered from systematic reviews or randomized controlled trials that validated herbal drugs for the management of osteoarthritic pain is weak. The purpose of this study is to explore the efficacy and safety of the *Shaoyao Shujin* tablet for the management of KOA in a short-term study.

**Methods/Design:**

This trial is a multicenter randomized, double-blind, placebo-controlled study. A total of 276 patients will be randomized into 3 groups: (1) the high-dose *Shaoyao Shujin* tablet group (HD group), (2) the low-dose *Shaoyao Shujin* tablet group (LD group), and (3) the placebo tablet group (control group). In the three groups, four tablets will be administered three times per day for 6 weeks. Follow-up will be at regular intervals during a 10-week period with the Western Ontario and McMaster Universities Index (WOMAC) score, visual analog scale (VAS) score, and rescue medication use assessed as outcome measures.

**Discussion:**

This study will provide clinical evidence on the efficacy and safety of the *Shaoyao Shujin* tablet in treating KOA.

**Trial registration:**

Chinese Cochrane Center ChiCTR-IPR-15006194, registered 4 April 2015.

## Background

Knee osteoarthritis (KOA) is a prevalent disease causing disability in the aging population. The risk of KOA disability, characterized as chronic pain and functional limitation, is as great as that attributable to cardiovascular disease and greater than that due to any other medical condition in older persons [1]. In the US, estimated average discounted (3 % per year) lifetime costs for persons diagnosed with KOA were US$140,300, and direct medical costs were US$129,600, with US$12,400 (10 %) attributable to KOA over 28 years [2]. Determining effective means of treating KOA has become an important subject of study, particularly because currently there is no medical treatment to reverse or stop its progression. Pharmacological approaches include analgesics, anti-inflammatory agents, corticosteroids given by the intra-articular route, or hyaluronic acid, glucosamine sulfate, and chondroitin sulfate, which are already widely use. However, these drugs have some adverse effects such as constipation, nausea, and excessive sedation in older people, and their effect on cartilage with OA changes remains controversial [3–5].

The aim of KOA treatment is to reduce pain, improve physical function, prevent disability, and enhance short-term quality of life. The herbal drug the *Shaoyao Shujin* tablet (SST, previously called *Yangxue ruanjian jiaonang*) is a multicomponent Chinese herbal supplement that has been proved to be an effective regimen to relieve KOA pain in patients [6] or to delay the degeneration of articular cartilage in KOA animal models [7, 8]. However, understanding of the mechanisms responsible for the beneficial effects of SST is limited and its clinical effectiveness needs strong evidence for proof. Published Chinese medical literature has documented that SST is efficacious in treating KOA, but these interventions have not been rigorously evaluated, and their credibility questioned, due to a lack of objective evidence [9]. Therefore, we designed a multicenter randomized, double-blind, placebo-controlled trial to explore the efficacy and safety of SST for the symptomatic management of KOA in a short-term study.

## Methods/Design

### Study design

This is a multicenter randomized, double-blind, placebo-controlled trial. General ethical approval was issued by the Ethics Committee of Guangdong Provincial Hospital of Chinese Medicine (No. A2015-01-2-1), and participating center ethical approval was issued by the Clinical Trial Ethics Committee of Huazhong University of Science and Technology (No. FZ-1302-01), the Ethics Committee of The First Affiliated Hospital of Anhui University of TCM (No. 2015AH-03), the Clinical Trial Ethics Committee of The First Affiliated Hospital of Hunan University of TCM (No. 2015-YW-001), and the Ethics Committee of Shanghai Hospital of TCM (No. 2015SHL-05). Subjects will be enrolled at these five hospitals. The trial protocol has been registered in the Chinese Clinical Trial Registry (ChiCTR-IPR-15006194). All study participants will sign the written informed consent before participation. The trial is financially supported by Shanghai Fosun Pharmaceutical Development Co., Ltd, Shanghai, China, which will participate in the study design, but will not be involved in data collection, data management, analysis, interpretation of data or the decision to submit the report for publication, but will provide all test drugs.

### Recruitment and consent

**Fig. 1:**
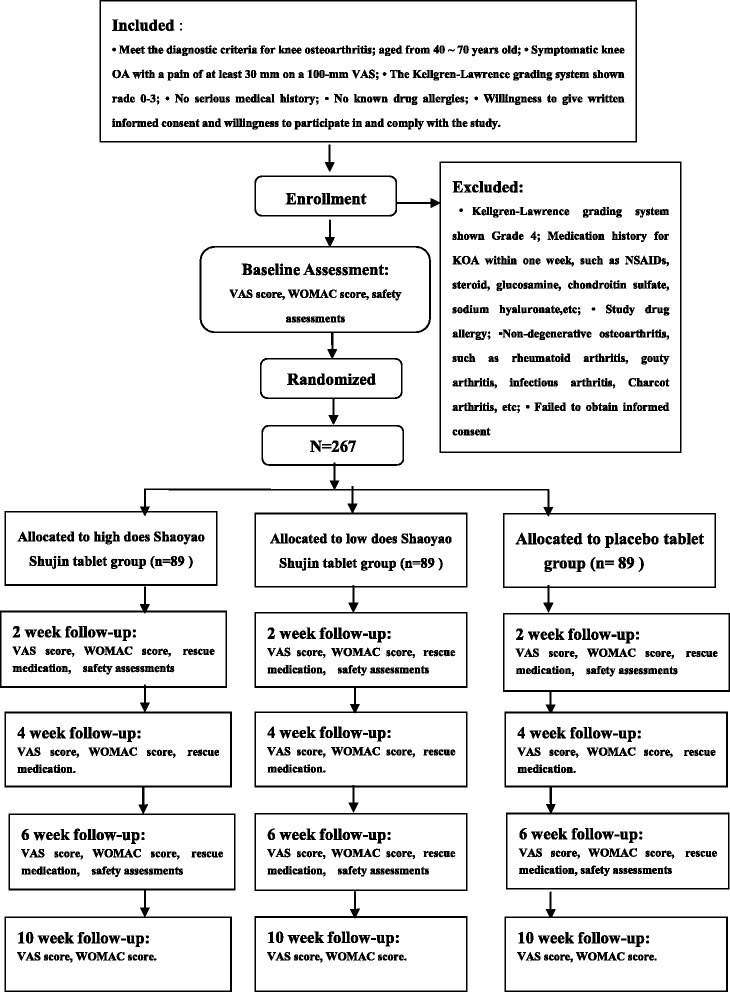
Study flow-chart

A target sample size of 276 KOA patients, who are regularly followed up at outpatient clinics of the Department of Orthopedic Surgery in each participating center, will be recruited. All candidates will go through a standardized interview process and receive more information about the study and the treatments. The purpose, procedures, and potential risks and benefits of the study will also be explained thoroughly to the participants. The more painful knee will be chosen as the study knee, and if both knees have the same symptoms, we will choose the right side. The participants will be able to withdraw from the study at any time without consequence. The trial will be executed from February 2015 to February 2016 including enrollment and follow-up (see Fig. 1).

### Inclusion criteria

Participants meeting the following criteria will be included:

- Meet the diagnostic criteria for KOA (American College of Rheumatology criteria [10])
- Aged from 40 to 70 years old
- Symptomatic KOA with pain of at least 30 mm on a 100-mm visual analog scale (VAS)
- Grade 0–3 on the Kellgren-Lawrence grading system
- No serious medical history
- No known drug allergies
- Willingness to give written informed consent and willingness to participate in, and comply with, the study

### Exclusion criteria

Participants meeting one or more of the following criteria will be excluded:

- Grade 4 on the Kellgren-Lawrence grading system
- Medication history for KOA within 1 week, such as NSAIDs, steroids, glucosamine, chondroitin sulfate, sodium hyaluronate, etc.
- Allergy to study drug
- Non-degenerative osteoarthritis, such as rheumatoid arthritis, gouty arthritis, infectious arthritis, Charcot arthritis, etc.
- Unwilling to give informed consent

### Interventions

Eligible patients will be randomized into the three groups: (1) the high-dose SST group (HD group), (2) the low-dose SST group (LD group), and (3) the placebo tablet group (control group). The SST will be provided by the Shanghai Fosun Pharmaceutical (Group) Co., Ltd, Shanghai, China (drug permit license number 2014L00464) and weighs 0.35 g per tablet. The components of the SST are *Radix paeoniae alba*, *Radix Gentianae Macrophyllae*, oyster, licorice, scorpion, and centipede. The placebo tablet will be manufactured identical to the SST in terms of color and odor by the same manufacturer. In the HD group four SSTs will be administered three times per day for 6 weeks, while in the LD group two SSTs and two placebo tablets, and in the control group four placebo tablets at the same frequency and for the same duration. Patient visits will be performed at baseline and 2, 4, 6 and 10 weeks after treatment. Assessments including the Western Ontario and McMaster Universities Index (WOMAC) score and the VAS score will be recorded on the relevant knee during all visits.

As a short-term study aims to explore the efficacy and safety of SST for the symptomatic management of KOA, we think that 6 weeks’ treatment and 4 weeks’ follow-up will be enough to observe whether there are differences between the HD group, the LD group and the placebo tablet group.

During the process, when patients experience moderate pain or a VAS score over 40 mm, ibuprofen sustained-release capsules will be administrated as rescue medication. Rescue medication consumption and time will be recorded in each randomized group when the subjects use rescue medication. The patients will not be allowed to use other drugs aiming to treat KOA.

### Outcome measures

#### Primary outcome measure

The primary efficacy endpoint of the study will be the WOMAC score [11]. The WOMAC score is a widely used proprietary set of standardized questionnaires used by health professionals to evaluate the condition of patients with osteoarthritis of the knee and hip, including pain, stiffness, and physical functioning of the joints. The index measures five items for pain (score range 0 to 20), two for stiffness (score range 0 to 8), and 17 for physical function (score range 0 to 68). It will be measured during all the assessment visits (baseline, 2-, 4-, 6- and 10-week follow-up).

#### Secondary outcome measure

The secondary efficacy endpoint of the study will be the VAS score, which is a pain score ranging from 0 mm (no pain) to 100 mm (worst pain ever experienced) [12], measured during all the assessment visits (baseline, 2-, 4-, 6- and 10-week follow-up). The VAS score is usually a horizontal line, 100 mm in length, anchored by word descriptors at each end. Patients mark the point of their current pain on the line. The VAS score is then determined by measuring in millimeters from the left end of the line to the point that the patient marked.

### Safety assessments

All subjects will be questioned about adverse events during the treatment at each visit, and all adverse events reported will be analyzed, regardless of the investigators’ assessments of causality. Safety will be assessed by the following tests on all subjects: physical examination (temperature, respiration, heart rate, blood pressure, height and weight), complete blood cell count, urinalysis, stool examination, fecal occult blood test, liver function (alanine aminotransferase (ALT), aspartate aminotransferase (AST), alkaline phosphatase (ALP), serum total bilirubin (STB), and γ-glutamyl transpeptidase (γ-GT)), renal function (creatinine (Cr), micro-albuminuria, serum cystatin C, and urinary *N*-acetyl-β-glucosaminidase) and an electrocardiogram (ECG) at baseline and at 2- and 6-week visits.

### Randomization and blinding

Patients who meet the inclusion criteria will be randomly assigned to one of three groups (HD group, LD group, control group) in a ratio of 1:1:1 using a computer-generated random allocation sequence through the stratified block randomization method of SAS version 9.1.3 (SAS Institute Inc., Cary, NC, USA). Each patient will receive a unique randomized test number corresponding to the specified drug, according to the group allocation. An emergency envelope has been prepared for each test number and is to be opened.

The trial will be blinded to both patients and treating physicians. For test drug blinding, the placebo tablet will be manufactured identical to the SST in terms of color and odor. The drugs are administered by an independent clinical assistant in each center who takes responsibility for the drugs’ distribution, storage and return. Any members who have access to the drug will not participate in case observations or efficacy evaluations. Before the trial two different bottles will be prepared: bottle A contains a 2-week dose of SSTs and bottle B contains a 2-week dose of placebo tablets. Bottles A and B will be packaged with the same label for patient and assessor blinding. For the HD group, two A bottles will be assigned; for the LD group, one A bottle and one B bottle will be assigned; and for the control group, two B bottles will be assigned. During baseline, and 2-, and 4-week visits, patients will be assigned two bottles according to their group, and 14 rescue medications. They will then be instructed to take two tablets from each bottle every time they take their drugs. At the next visit, the patient should return all the rescue medications they didn't taken.

### Sample size

Calculation of sample size is based on a similar herbal drug study assessing the short-term efficacy of two types of Traditional Chinese herbal patches, *Fufang Nanxing Zhitong Gao* and *Shangshi Jietong Gao* [13]. We estimate that an absolute improvement of 10 in the WOMAC total score is likely to be the smallest clinically relevant difference for patients with KOA. We assume that the standard deviation of the total WOMAC score to be 18.2 at baseline. Based on these assumptions, we will require 79 patients in each group to have at least an 80 % power (*β* = 0.8) and to rule out a two-sided type I error of 5 % (*α* = 0.05). The number of patients actually provides less than 80 % power, assuming a withdrawal rate of 15 %. Therefore, we will recruit a total of 276 patients, 92 patients in each group.

### Statistical analysis

The data will be collected and analyzed according to the intention-to-treat principle. An analysis of variance (ANOVA) or chi-square test will be used to compare baselines among patients. Efficacy analyses will be performed for both the intent-to-treat (ITT) population and the per-protocol (PP) population. The ITT population will consist of all randomized subjects who have been administered at least one treatment. In a PP analysis, only patients who complete the entire clinical trial according to the protocol are counted towards the final results. Primary outcome will be compared among groups; the extent and time pattern of rescue medication use in each randomized group will be reported: in considering comparison of the underlying outcomes of rescue medication use, we will assume the use varies with time and, to allow for dependence within patients, we will adopt a multilevel regression approach with rescue as a time-dependent covariate [14]. All statistical analyses will be performed using SAS 9.2 software. All statistical tests will be two-sided, and the level of significance will be set at 0.05.

## Discussion

This study will provide clinical evidence on the efficacy and safety of SST in treating KOA. Despite considerable research, there is no specific remedy for patients who suffer from OA. Various complementary and alternative medical treatments have been administered for OA in clinical practices [15–17]. Some studies have reported the effectiveness of herbal medicine, which is the most popular form of complementary and alternative medical therapy for the treatment of OA [18–21]. Insects have also been used globally as traditional medicines for centuries [22]. Several studies have shown that leeches have a remarkable effectiveness in treating osteoarthritis pain [23–25]. As a Traditional Chinese Medicine (TCM) formula, SST, containing herbal medication and insects, has been demonstrated to be effective at activating blood circulation and assuaging pain in previous clinical observation. However, due to lack of objective evidence, a well-designed randomized controlled trial is needed to examine the efficacy and safety of SST for the symptomatic management of KOA.

## Trial status

Recruitment will commence in February 2015, and the trial will schedule to end in February 2016.

## Abbreviations

ALP: alkaline phosphatase
ALT: alanine aminotransferase
AST: aspartate aminotransferase
Cr: creatinine
ECG: electrocardiogram
γ-GT: γ-glutamyl transpeptidase
ITT: intent-to-treat
KOA: knee osteoarthritis
PP: per-protocol
TCM: Traditional Chinese Medicine
SST: *Shaoyao Shujin* tablet
STB: serum total bilirubin
TCM: Traditional Chinese Medicine
VAS: visual analog scale
WOMAC: Western Ontario and McMaster Universities Index
